# Why does frugality influence the recycling intention of waste materials?

**DOI:** 10.3389/fpsyg.2022.952010

**Published:** 2023-01-20

**Authors:** Hong Wang, Rubing Bai, Haibo Zhao, Zhichen Hu, Yan Li

**Affiliations:** ^1^School of Economics and Management, Beijing University of Agriculture, Beijing, China; ^2^School of Management and Economics, Beijing Institute of Technology, Beijing, China; ^3^Business School, Shandong University, Weihai, China

**Keywords:** frugality, recycling intention, environmental concern, perceived value, waste recycling

## Abstract

Waste recycling significantly impacts the sustainable development of society and the ecological environment, contributing to a vital role within the waste management hierarchy. This paper presents a research model that investigates the influence mechanism of consumers’ frugality on their recycling intentions. This study collected 420 valid samples to test the model with regression analysis. The empirical results show that consumers’ frugality exerts a direct and positive effect on their recycling intention. Except for the positive direct effect, perceived value mediates the relationship between frugality and recycling intention. Besides, environmental concern strengthens the positive relationship between frugality and recycling intention. The findings of this study can better explain the recycling intention, thereby providing a basis for the government and enterprises to formulate policies and measures to promote recycling behavior.

## 1. Introduction

With the economy’s rapid growth, China has become the largest consumer market in the world. Meanwhile, a lot of household waste is produced every day. For example, an average of 2.14 kg of municipal solid waste was generated daily by every Hong Kong resident in 2015, and 1.39 kg was discarded into the landfill (Environmental Protection Department, 2016; Legislative Council Secretariat, 2017). Two-thirds of China’s large and medium-sized cities are reportedly submerged in garbage, with more than 500 million square meters of land invaded nationally due to solid waste (Fei et al., 2016). According to estimations, the amount of municipal solid waste produced globally may rise from 2.01 billion tonnes in 2016 to 3.40 billion tonnes in 2050 and 3.83 billion tonnes in 2100 (World Bank, 2018), of which around 70% will not be recycled (Wilson et al., 2015). Over the previous several decades, the amount of different waste streams has increased quickly, and total municipal solid waste amounts are predicted to reach 480 million metric tons in 2030, with an average growth rate of 8–10% since 2000 (Chu et al., 2019). Poorly managed municipal solid waste may threaten human health and the natural environment. The appropriate disposal of waste is a challenge for city authorities and governments (Xu et al., 2017). The government has introduced various incentive measures to promote individual recycling behavior (Wilson et al., 2012; Tencati et al., 2016). Recycling is an effective means of sustainable urban development, which can turn waste into treasure (Rhodes et al., 2015; Marino et al., 2018). It can not only save natural resources but also reduce the impact on the environment and the demand for landfill (Chen and Tung, 2010). Meanwhile, President Xi Jinping clearly emphasized the need to establish waste classification scheme for more regions when he chaired the Central Leading Group on Financial and Economic Affairs in December 2016. The National Development and Reform Commission and the Ministry of Housing and Urban–Rural Development issued the “Household Solid Waste Classification System Implementation Plan” on March 18, 2017, specifying that 46 cities across the country adopt the required sorting of solid waste and that recycling rates surpass 35% by 2020. Although household recycling has been considered essential and developed in recent years, the present recycling management is not satisfactory, and household participation in waste separation remains low (European Environment Agency, 2016; Yan, 2018). The rise of urban garbage is a severe issue, and it hinders residents’ health and ecological environment (Knickmeyer, 2020). It is a pressing issue to provoke consumer participation in terms of recycling waste. To improve the recovery rate of waste, it is necessary to excavate the primary influencing factors of recycling to enhance people’s recycling participation, which has significant theoretical and guiding significance.

Previous studies have investigated the factors that influence recycling intention (Paola et al., 2019; Yu et al., 2019; Yue et al., 2020). Such as, sociodemographic factors (Wang et al., 2018a), socioeconomic factors (Xu et al., 2017; Wang C. et al., 2021) and psychological factors (Calvin et al., 2021; Wang Q. et al., 2021; Emmanuel et al., 2022). However, personality trait is the fundamental determinant of human behaviors (Furnham and Heaven, 1999; Swami et al., 2011). Frugality is one of the characteristics of personal consumption behavior and is considered to be closely related to sustainable consumption behavior. Thus, this paper aims to investigate whether and how frugality influences people’s recycling intentions. The main contribution of this paper is to increase understanding of the association between frugality and recycling behavior, and guide consumers to establish environmentally friendly behavior.

The rest of the paper is structured as follows. We display the theoretical basis and hypotheses development in Section 2. The study shows the research method in Section 3. Section 4 analyzes the data and presents the statistical results. Section 5 reveals the research conclusions and discussions. We give the management implications and limitations in Section 6.

## 2. Theoretical basis and hypotheses development

### 2.1. Theoretical basis

Frugality has been defined as “a unidimensional consumer lifestyle trait characterized by the degree to which consumers are both restrained in acquiring and resourceful in using economic goods and services to achieve longer-term goals” (Lastovicka and Hughner, 1999). There are many forms of frugality, and different scholars have a different understanding of frugality. Some researchers argue that frugality may be a lifestyle trait (Lastovicka and Hughner, 1999), a single value orientation (Bearden et al., 2006), and a behavior model (Egol et al., 2010). Normally, frugal consumers have three features (Lastovicka and Hughner, 1999). First, in their expenditure, frugal consumers are more self-restrained and pay more interest to long-term gains. Second, frugal people do their best to maximize the benefit of their assets. Third, frugal consumers are hardly impacted by social influences than ordinary consumers.

With the improvement of the urban economy and living standards, the amount of household waste increases at an alarming rate. The landfill is a way to solve household waste, but it not only pollutes the environment seriously but also reduces the available land area. Recycling is conducive to cut down the demands for valuable landfill space (Environmental Protection Department, 2010) and transforms waste into useful resources (Chen and Tung, 2010). Recycling can generate a great many environmental, economic, and social benefits. Previous studies used the theoretical model to analyze people’s recycling intentions, such as theory of planned behavior (Mahmud et al., 2020; Calvin et al., 2021; Boqi and Jianping, 2022), norm activation model (Wang et al., 2018b; Emmanuel et al., 2022), institutional theory (Sourabh et al., 2020), self-determination theory (Cho, 2019). Some scholars analyze recycling behavior from selfless motivation and altruistic nature (Botetzagias et al., 2015; Ofstad et al., 2017), individuals’ moral considerations (Tonglet et al., 2004; Chen and Tung, 2010), psychological factors (Hung and Fen-Hauh, 2015; Han et al., 2018) and environmental factors (Sujata et al., 2019; Manuel et al., 2022). In addition to the above factors, Refsgaard and Magnussen (2009) and Pei (2019) suggested that recycling activity relies on technological, organizational, and institutional aspects. In addition, some scholars explain environmental friendly behavior from personal values because personal values are the basis of people’s behavior patterns (Follows and Jobber, 2000; Roccas et al., 2002; Do Valle et al., 2005). Similarly, personality traits are the basic determinant of people’s behaviors (Furnham and heaven, 1999; Swami et al., 2011). Frugality is a characteristic of human beings. There are few studies on the influence mechanism of frugality on recycling intention. Consumers with a higher frugal consumption concept have less materialism and purchase less. As for the waste products, they generally seek to maximize utility of the products through repair and reuse (Albinsson et al., 2010), and then increase the service life of products. They also prefer to tap the potential value of their products. At the end of the product life cycle, consumers’ frugality may affect their recycling behavior. Namely, consumers with high frugality are more willing to conduct recycling behavior.

### 2.2. Hypotheses development

Frugality is regarded as the careful use of resources to avoid waste. Frugal behavior is usually praised and encouraged as a well-recognized value and a good way of life. Consumers with a strong sense of frugal consumption pursue the maximization of product use value (Albinsson et al., 2010) and extend product service life. They try to maximize the utility of the product as much as possible within the service life of the product. Moreover, frugal consumers aim to optimize the value of money when they consume and avoid unnecessary extravagance. In the use of products, they pursue the maximum utility of products and are resourceful about their products, which increases their likelihood of recycling.

In addition, perceived value has garnered a great deal of interest in consumer behavior research in recent years. Zeithaml (1988) defined that it is the perspective of a consumer’s total assessment of a product (or service). It might be seen as a balance between perceived benefits and perceived costs. In this study, “perceived value” can be considered of as the residents’ overall perception after balancing the perceived benefits of recycling and the perceived costs of recycling. Perceived value is an important predictor of future behavior. It can be inferred that that residents’ perceived value plays a critical predictive role in their recycling intention. The higher the residents’ perceived value is, the higher their recycling intention will be. At the end of the product life cycle, frugal consumers exert the surplus value of the product as much as possible. The surplus value of the product enhance residents’ perceived value of recycling and further promote residents’ participation intention in recycling. Recycled products may increase the income of residents, which is also in line with the saving characteristics of frugal consumers. Based on theory of reasoned action, we can speculate that frugal consumers may take the initiative to explore the potential value of the product and increase their willingness to conduct recycling behavior. Based on the above analysis, we hypothesized that:

*H1*: Frugality is positively associated with recycling intention.

*H2*: The relationship between frugality and recycling intention is mediated by perceived value.

Environmental concern indicates the degree to which individuals’ awareness of environmental issues and their willingness to address them (Dunlap and Jones, 2002). Environmental concerns reflect consumers’ views on environmental issues and their strong attitudes towards environmental protection (Crosby et al., 1981; Chan and Lau, 2000). Consumers with deep ecological concerns might have a high sense of ecological obligation and are more inclined to engage in environmental activities to protect the environment (Ramayah et al., 2010; Biswas and Roy, 2015; Pham et al., 2019). They tend to integrate the concept of environmental protection into their lives. That is to say, environmental concern plays a crucial function in encouraging environmental behavior (Wang et al., 2017; Molinillo et al., 2020). Similarly, Kushwah et al. (2019) found evidence for the moderating effect of environmental concern on connections between consumer value and environmentally friendly behavior.

Additionally, recycling waste is considered to reflect behavior that is environmentally protective and promotes sustainability. Consequently, we believe that frugal consumers with high environmental concerns pay more attention to environmental issues in their lives and have a stronger attitude of pro-environmental behavior. They have high environmental concerns and are more likely to transform their ecological responsibility into recycling behaviors. Thus, we posed the following hypothesis:

*H3*: The relationship between frugality and recycling intention is positively moderated by environmental concern.

**Figure 1:**
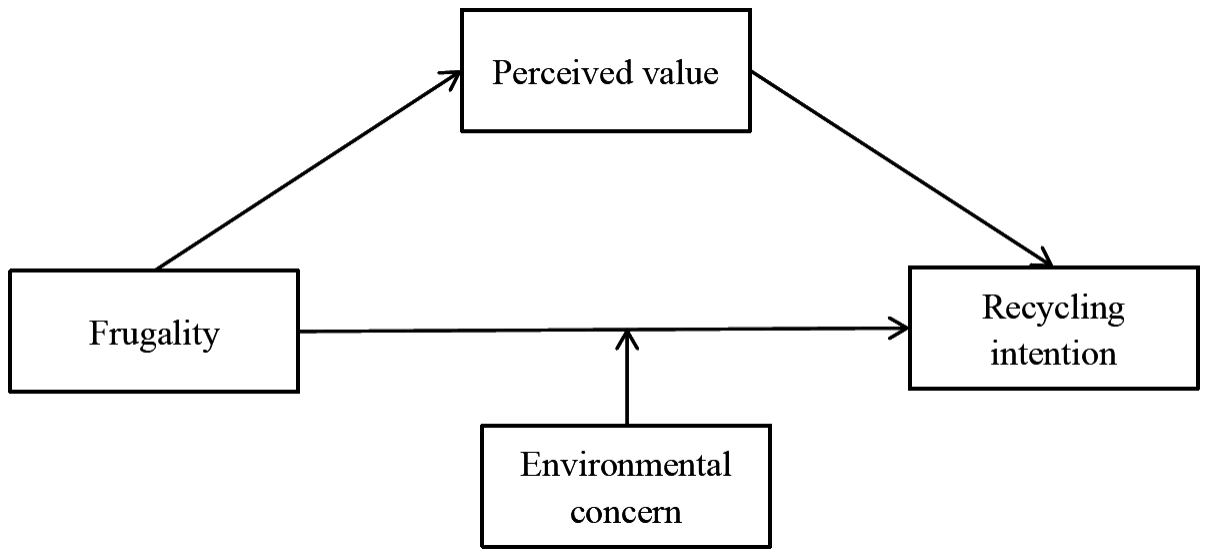
Conceptual framework.

Drawing upon the above literature and analysis, a personality-perception-intention framework was introduced to analyze the connection between frugality and recycling intention. We developed perceived value based on the theory of value. Figure 1 presents the conceptual framework.

## 3. Methodology

### 3.1. Sample and data

The questionnaire survey was sent out through Sojump.^1^ Respondents can obtain a monetary incentive to fill the survey. After deleting the invalid questionnaires, 420 valid questionnaires were analyzed. The useful questionnaires include respondents from different demographic backgrounds. The demographic details are shown in Table 1. Female made up 51.9 of all participants. The majority of participants were aged between 20 and 40 and held bachelor and Junior college degrees. Most of the respondents were staff, and monthly income was below 10,000 yuan for 93.1% of respondents.

**Table 1:** Demographic characteristics of the sample.

Variable	Categories	Frequency	Percent (%)
Gender	Male	202	48.1
	Female	218	51.9
Age	≤20	11	2.6
	21–30	198	47.1
	31–40	149	35.5
	41–50	47	11.2
	≥51	15	3.6
Education	High school or below	18	4.3
	Junior college degree	112	26.7
	Bachelor’s degree	212	50.5
	Master’s degree or above	78	18.6
Employment	Student	109	26.0
	Working	298	70.9
	Others	13	3.1
Monthly income(CNY)	≤3,000	104	24.8
	3,001–6,000	146	34.8
	6,001–10,000	141	33.6
	>10,000	29	6.9

### 3.2. Measures

In order to ensure the validity of the items, this study adopted the mature scale of previous studies. Items for frugality were derived from Evers et al. (2018). Items for perceived value were developed based on Sirdeshmukh et al. (2002) and Parasuraman (1997). The items of environmental concern were adapted from Mostafa (2009) and Yadav and Pathak (2016). The measurement of recycling intention was adapted from a prior study Yue et al. (2020) and Wan et al. (2017). According to the research background, we adapted the scales to meet the needs of this study.

Due to the initial items were in English, a back-translation process was used to transform English items into Chinese. Based on the research context, we revised the items. Then, we invited five students to review the questionnaire. Based on their comments, we made some modifications to the items. The final version of the questionnaire is easily understood from the standpoint of the respondents. The study used 7-point Likert-type to measure the items, ranging from strongly disagree (1) to strongly agree (7).

## 4. Data analysis and results

We analyzed the survey data with SPSS 27 and AMOS 20. First, the reliability and validity of the measurement model were examined. Second, all hypotheses were tested in this study.

### 4.1. Measurement model

Before testing the hypotheses, we performed confirmatory factor analysis (CFA) to assess the reliability and validity of the measurement model. Table 2 shows the reliability and validity analysis results of the scales. The Cronbach’s alpha of each construct ranged from 0.770 to 0.901. Thus, it can be inferred that the scale is highly reliable (Fornell and Larcker, 1981; Nunnally and Bernstein, 1994). Furthermore, the composite reliability scores of all constructs were greater than 0.7. For each construct, the average variance extracted (AVE) surpassed the cut-off value of 0.5. Given the above analysis, the scale achieves enough convergent validity (Bagozzi and Yi, 1988).

**Table 2:** Results of measurement model analysis.

Construct	α	CR	AVE
Frugality	0.806	0.811	0.524
Perceived value	0.901	0.898	0.746
Environmental concern	0.770	0.769	0.625
Recycling intention	0.874	0.879	0.708

α = Cronbach’s alpha; CR = composite reliability; AVE = average variance extracted.

After testing the convergent validity, discriminant validity was checked in this study. Table 3 displays the results of discriminant validity analysis. It can be seen that the square root of AVE for each construct was greater than its correlation with other constructs. Thus, the discriminant validity of the scale was ensured (Paulraj et al., 2008).

**Table 3:** Discriminant validity analysis.

Constructs	FR	PV	EC	RI
Frugality(FR)	0.724			
Perceived value (PV)	0.255	0.864		
Environmental concern (EC)	0.162	0.285	0.791	
Recycling intention (RI)	0.391	0.346	0.335	0.841

1. Off-diagonal elements are correlations between constructs; 2. Diagonal elements are the square root of average variance extracted.

Since the measurements are derived from self-reported data, the single data survey may result in common method bias. Harman’s single-factor test was used to analyze the possibility of bias (Podsakoff et al., 2003). The results reported that four factors were extracted, and the maximum explained variance of the factor was 34.8% below the recommended threshold of 50%. Thus, it can be inferred that the common method bias was impossible to be a concern in current study (Malhotra et al., 2006).

### 4.2. Hypothesis testing

To measure the mediating effect and moderating effect concurrently, this study implemented hierarchical regression to analyze the relationships between variables.

#### 4.2.1. Main effect

Table 4 presented the results of hierarchical regression. Model 1 showed the connection between control variables and the dependent variable. Based on Model 1, the independent variable was added into Model 2, and the result indicated that the positive influence of frugality on recycling intention is established (*β* = 0.374, *p* < 0.001). Hence, hypothesis H1 is verified in the study.

**Table 4:** Hierarchical regression result.

Dependent variables	Recycling intention		Perceived value	Recycling intention		
	M1	M2	M3	M4	M5	M6
1. Control variables						
Gender	0.393^***^	0.394^***^	0.222	0.331^**^	0.344^***^	0.393^***^
Age	−0.102	−0.033	−0.031	−0.08	−0.026	−0.061
Education	−0.032	−0.018	0.156	−0.073	−0.054	0.001
Employment	−0.033	−0.027	0.032	−0.041	−0.035	−0.042
Income	−0.071	−0.096	−0.084	−0.052	−0.077	−0.109
2. Independent variable Frugality		0.374^***^	0.261^***^		0.315^***^	0.334^***^
3. Mediating variable Perceived value				0.281^***^	0.226^***^	
4. Moderating variable Environmental concern						0.311^***^
5. Moderating effect						
Frugality * Environmental concern						0.087^*^
R^2^	0.042	0.156	0.065	0.139	0.216	0.226
Adj. R^2^	0.031	0.144	0.051	0.126	0.202	0.211
F	3.646^**^	12.713^***^	4.758^***^	11.077^***^	16.178^***^	14.993^***^

*p < 0.05; **p < 0.01; ***p < 0.001.

#### 4.2.2. Mediating effect

We adopted the mediation analysis procedure from the study of Baron and Kenny (1986). The study tested the mediation effect of perceived value in four steps. Model 2 demonstrated that the impact coefficient of frugality achieves a significant level. Model 3 suggested the regression model of frugality on perceived value, and the result reported that frugality significantly influences perceived value (*β* = 0.261, *p* < 0.001). Model 4 exhibited the regression model of perceived value on recycling intention, and the result displayed that perceived value is positively associated with recycling intention (*β* = 0.281, *p* < 0.001).

In view of Model 2, Model 5 introduced the mediating variable of perceived value, and the result exhibits that frugality is still positively associated with recycling intention (*β* = 0.315, *p* < 0.001). The perceived value is positive correlation with recycling intention (*β* = 0.226, *p* < 0.001). According to the above analysis, perceived value partially mediates the relationship between frugality and recycling intention. Thus, the finding supports H2.

#### 4.2.3. Moderating effect

Three procedures were carried out to test the moderation analysis. First, to remove the differential contribution of variables due to measurements from various scales, we standardized the independent variable and the mediating variable, and then calculated an interaction item between the two variables. Second, the measurement variables were orderly added into the regression equation. Third, we introduced the interactive item into the regression equation.

In Model 6, it can be observed that environmental concern positively moderates the connection between frugality and recycling intention, and strengthens the positive effect between them (*β* = 0.087, *p* < 0.05). Therefore, Hypothesis H3 is supported.

**Figure 2:**
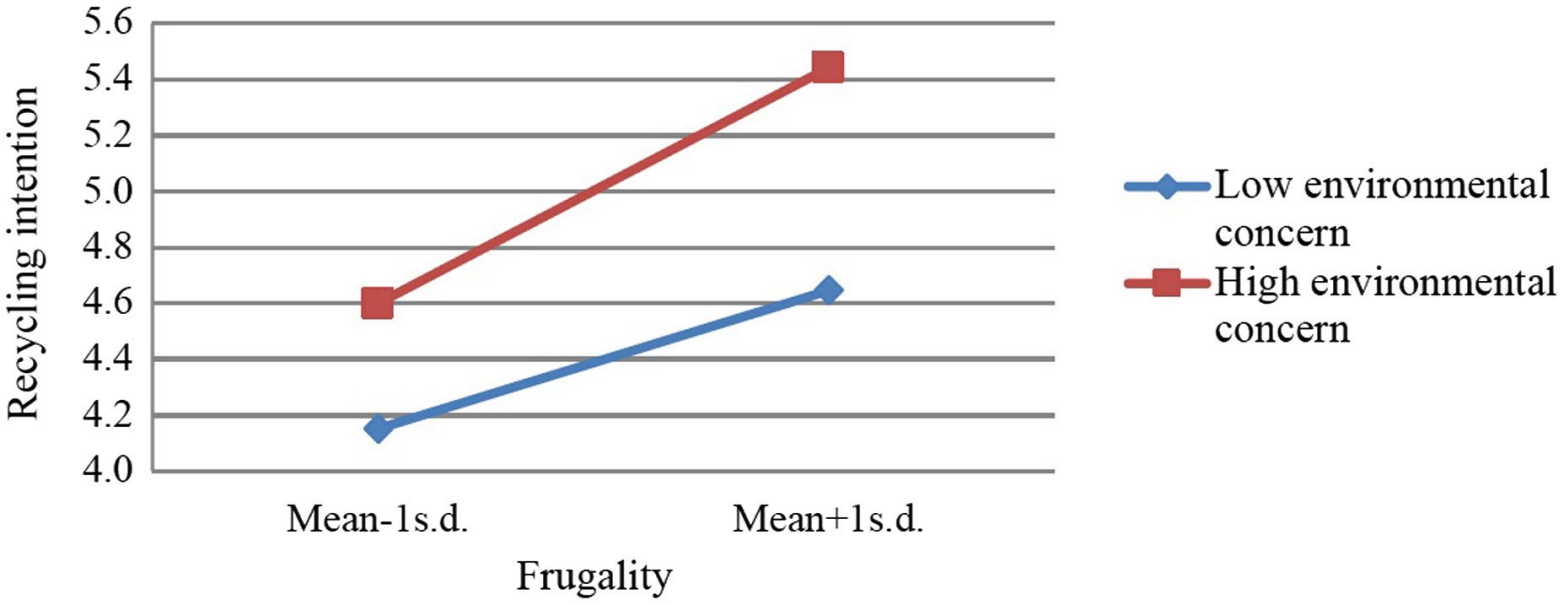
The moderating effect of environmental concern on the relationship between frugality and recycling intention.

This study further analyzes the moderating effect of environmental concern. We constructed high and low levels based on one standard deviation above and below the mean of environmental concern, and then depicted the interactive relationship (Li and Tang, 2010; Dawson, 2014). As shown in Figure 2, the connection between frugality and recycling intention was significantly influenced by the moderator of environmental concern.

## 5. Conclusion and discussions

### 5.1. Conclusion

This research aimed to examine the impact mechanism of frugality on recycling intention. After the data analysis, the results revealed that frugality is positively related to recycling intention. The influence of frugality on recycling intention is partially mediated by perceived value. The effect between frugality and recycling intention is positively moderated by environmental concern.

### 5.2. Discussions

This paper contributes to the research on people’ recycling participation intentions. Current studies on recycling behavior are mostly focused on TPB and other theoretical models to investigate the influencing factors of people’ recycling intention, and the link between individual behavior characteristics and recycling behavior has received little attention. This research demonstrates the influence mechanism of frugality on recycling intention, combining perceived value and environmental concern to investigate the process and context by which frugality affects recycling intention. It compensates for the deficiencies of previous recycling studies and discloses the law of citizens’ behavioral intention to recycle. In addition, existing studies have demonstrated that participation rate of recycling waste is still low, this study reveals what kind of people are willing to engage in the recycling program.

More specifically, this study illustrates the following findings.

First, frugality is positively related to recycling intention. Previous studies have tested the antecedents of frugal behavior (Suárez et al., 2020; Gil-Giménez et al., 2021). As an extension of previous studies (Muiños et al., 2015), the current study examines the follow-up behavior of frugality. Frugal consumers have the habit of saving resources and making the best of their resources. Consumers who have a strong sense of frugality pursue the maximum utility of product and are resourceful about their possessions (Albinsson et al., 2010). At the end of the product life cycle, frugal consumers try to make use of the surplus value of the product and pursue the maximization of its value to increase the recycling behavior.

Second, the influence of frugality on recycling intention is partially mediated by perceived value. Perceived value can be used as a mediator variable, which is consistent with previous studies (Wang C. et al., 2021; Wang Q. et al., 2021). Perceived value is an important factor driving personal behavior. The perceived value of waste determines whether frugal consumers recycle waste products. If waste products can be transformed into other useful resources or have the value of recycling, frugal consumers are likely to consider what to do with their waste products. Therefore, faced with waste products, frugal consumers are likely to be motivated by an awareness of residual value, thus increasing the recycling behavior of waste products.

Third, the effect between frugality and recycling intention is positively moderated by environmental concern. The effect of environmental concern, as a moderating variable, is consistent with previous studies (Zhang et al., 2018). Environmental concern demonstrates consumers’ views on their responsibility for the environment, making them realize their role in alleviating environmental issues. Their attitudes and ideas about the environment are reflected in actual environmentally friendly behaviors. Besides, consumers with significant environmental concerns know more adverse effects of waste products on the environment and are more inclined to participate in the recycling program. Therefore, at the end of the product life cycle, frugal consumers with a high environmental concern may be more likely to develop the surplus value of waste products and actively participate in recycling.

## 6. Implications and limitations

### 6.1. Practical implications

This paper has several practical implications. First, this research finds that frugal consumers are likely to increase the recycling behavior of waste products. Enterprises and retailers should publicize waste disposal methods through various media. Enterprises can judge customers’ consumption characteristics and behavior patterns through big data analysis (data accumulated through customer loyalty program), and screen out frugal customers and encourage them to join waste recycling programs. More importantly, when the appeal for recycling has not achieved good results, government should advocate the values of frugality and encourage people to develop a frugal lifestyle and consumption style. Besides, the government can also set a good example of frugality and call on people to learn from it. This is conducive to the formation of learning atmosphere and the establishment of frugal ideology for the whole society.

Second, the perceived value of waste determines people’s willingness to participate in recycling projects. In daily life, the publicity of residual value of waste products is easy to be generally ignored by the government and enterprises. Product advertisements should emphasize residual value of waste products instead of environmental issues. Enterprises should exhibit various forms of residual value on waste products and introduce to consumers how to make full use of the residual value of products through advertisements. This helps people fully understand the recycling value and the impact of recycling on the environment, so as to increase consumers’ recycling knowledge and willingness to participate in recycling programs. Enterprises can try to implement the old for new and waste recycling points exchange way, enhancing the willingness of consumers to recycle. Moreover, the government should publicize the surplus value of waste products and the impact of waste products on the environment (e.g., public service advertisement), and report the specific data of annual resource saving through waste recycling. This contributes to increase people’s awareness of recycling, and stimulate people to develop frugal living habits and internalize the saving concept.

Third, environmental concern can strengthen the association between frugality and recycling intention. Consumers with high environmental concern have a better ability to figuring out the real environmental impact of products, and have a stronger sense of environmental issues. This drives them to engage in environmentally sustainable practices and recycle programs with a deep understanding of environmental obligation (Bamberg, 2003). Moreover, When people obtain details on why they should participate in such particular ethical actions, individuals would be more inclined to behave in altruistic ideals (De Groot and Steg, 2008). Therefore, enterprises should develop information strategies and communicate environmental awareness and knowledge among consumers. The marketers should introduce the environmental attributes and surplus value of products to attract consumers’ attention, stimulating their environmentally friendly behavior (Pagiaslis and Krontalis, 2014). In addition, the government should make efforts to provide recycling facilities, and play a leading role in promoting people’s recycling knowledge and environmental education through multiple effective ways of communication (Jaiswal and Kant, 2018). The government could also deliver the information concerning pollution generated from waste product and how much pollution can be minimized by recycling waste. In the long run, the subtle influence of government publicity can gradually stimulate frugal consumers’ recycling habits and behaviors.

### 6.2. Limitations and future research

Several limitations should be noted in this study. First, the results of this study are based on Chinese samples. People’s behavior is easily affected by social environment. There are differences in culture between different countries and people’s way of thinking and behavior. For example, Chinese consumers habitually save money for consumption mode, while European and American consumers focus on excessive consumption. Therefore, there is a need to replicate this research to other countries.

Second, the study is limited to measuring recycling intention rather than recycling behavior. Although behavioral intention is strongly associated with actual behavior (Tan and Teo, 2000; Hung et al., 2003), we should distinguish between two variables. There is a gap between behavior intention and real behavior. To be more specific, behavior intention may not be able to transform into real behavior. In the daily life, people’s behavior will be affected by environmental factors. Thus, it is necessary that we study recycling behavior in the future research.

Third, in China, different cities have different progress in introducing recycling policies. For example, first-tier cities introduced recycling policies earlier than other cities. The recycling policy issued by the government has a subtle impact on people’s behavior, thus people have a higher willingness to participate in the recycling project. Therefore, it is necessary to further explore the difference of people’s recycling intentions in different cities.

## Data availability statement

The raw data supporting the conclusions of this article will be made available by the authors, without undue reservation.

## Ethics statement

Ethical review and approval were not required for the study on human participants in accordance with the local legislation and institutional requirements. Written informed consent for participation was not required for this study in accordance with the national legislation and the institutional requirements.

## Author contributions

HW and HZ conceived the study and wrote the first draft of the article. ZH and YL were responsible for data collection. RB and YL were responsible for revising and proofreading the manuscript. All authors contributed to the article and approved the submitted version.

## Funding

This study was supported by Program for Scientific Research and Innovation Ability Improvement of Young Teachers, Beijing University of Agriculture (Project No. QJKC-2022056) and National Social Science Fund of China (Project No. 22BGL200).

## Conflict of interest

The authors declare that the research was conducted in the absence of any commercial or financial relationships that could be construed as a potential conflict of interest.

## Publisher’s note

All claims expressed in this article are solely those of the authors and do not necessarily represent those of their affiliated organizations, or those of the publisher, the editors and the reviewers. Any product that may be evaluated in this article, or claim that may be made by its manufacturer, is not guaranteed or endorsed by the publisher.

## Footnotes

^1^ http://www.sojump.com/
